# Neurological update: hereditary neuropathies

**DOI:** 10.1007/s00415-022-11164-1

**Published:** 2022-05-21

**Authors:** Caroline Kramarz, Alexander M. Rossor

**Affiliations:** Department of Neuromuscular Disease, Queen Square UCL Institute of Neurology and the National Hospital of Neurology and Neurosurgery, London, WC1N 3BG UK

**Keywords:** Charcot–Marie–tooth disease, SORD-associated CMT, PMP22 gene silencing, SPTLC1-associated HSN1, ATTR amyloidosis

## Abstract

In this update, we review the recent discovery of autosomal recessive variants in sorbitol dehydrogenase as one of the commonest and potentially treatable causes of hereditary motor neuropathy and CMT2. We also report on recent therapeutic advances in hereditary neuropathy including the use of lipid nanoparticle sequestered antisense oligonucleotides in CMT1A and lipid nanoparticle delivered CRISPR-Cas9 gene editing in ATTR amyloidosis.

## Introduction

The field of hereditary neuropathy, more commonly referred to as Charcot–Marie–Tooth disease (CMT) has made significant progress in recent years with respect to both gene discovery and treatment. In this review, we report on the recent discovery of recessive mutations in sorbitol dehydrogenase as one of the most common and potentially treatable causes of axonal CMT (CMT2) [1] and how advances in genetic discovery have expanded the phenotypes attributable to individual genes, none more so than dominant mutations in *SPTLC1* that are known to cause HSN1 but in which a new set of mutations now give rise to ALS [2, 3]. Finally, we draw on the emerging field of therapeutics that would have been unimaginable only 10 years ago, from a nutritional therapeutic strategy for HSN1 [4, 5] through to gene silencing therapy for CMT1A [6] and the emergence of gene editing using CRISPR Cas-9 in ATTR [7].

## (*SORD*)-associated CMT

To date, more than 100 causative genes have been identified to cause CMT and the process by which genetic testing is undertaken has been revolutionised by next-generation sequencing. Despite these advances, only 15 to 32.5% of patients with dHMN receive a genetic diagnosis [8]. A recent study by Cortese et al. [1] has identified sorbitol dehydrogenase (*SORD*) gene mutations as the most common cause of recessive axonal distal motor neuropathy accounting for up to 10% of undiagnosed cases of dHMN and CMT2. The emergence of *SORD*-associated CMT neuropathy comes with promising therapeutic intervention in the form of aldose reductase inhibitors.

Using a large cohort of over 1000 CMT patients, Cortese et al. identified biallelic pathogenic variants in *SORD* in 45 cases with CMT2 or dHMN, the majority of which carried the p.Ala253GlnfsTer27 in homozygous or compound heterozygous form.

The clinical phenotype was a slowly progressive, length-dependent, predominantly distal motor axonal neuropathy. The majority of cases were sporadic (69%) and the mean age of onset was late adolescence with 50% of cases reporting sensory symptoms. Serum fasting sorbitol concentrations for patients homozygous for p.Ala253GInfsTer27 were increased tenfold, compared to controls. Additional case series from the Czech Republic and China has confirmed *SORD* neuropathy to be one of the commonest causes of dHMN and CMT2 [9, 10].

*SORD* mutations lead to a reduced level and impaired function of the *SORD* enzyme, resulting in accumulation of sorbitol in blood and tissue [11]. *SORD* is involved in the second step of the polyol pathway; the initiating step of which is the conversion of glucose to sorbitol by aldose reductase, followed by *SORD*-mediated oxidation of sorbitol to fructose. This pathway has been implicated in the pathophysiology of preclinical models of diabetic neuropathy [12] and aldose reductase inhibitors have been shown to be effective in reducing sorbitol accumulation in mouse models of diabetes, and in humans. Cortese et al. demonstrated their use significantly reduced and normalised sorbitol levels in patient fibroblasts. Epalrestat, currently approved in several countries and Ranirestat, still in trial, are pharmacological aldose reductase inhibitors used to prevent disease progression in diabetic neuropathy and have a favourable safety profile [1]. This represents a possible treatment option for patients suffering with *SORD*-associated CMT.

## Serine palmitoyltransferase, long-chain base subunit-1 (SPTLC1)-associated HSN1: a new phenotype

The hereditary sensory neuropathies (HSN) represent a phenotypic extreme of CMT2 presenting with progressive length-dependent small-fibre impairment [13]. The marked sensory loss leads to severe attacks of pain, skin ulceration, infection, and injury [14].

Hereditary sensory neuropathy type 1 (HSN1) is the most common subtype with an autosomal dominant pattern of inheritance, caused by several missense mutations in *SPTLC1* [15, 16], a subunit of the enzyme serine palmitoyltransferase (SPT) [17]. SPT catalyses the pyridoxal-phosphate dependent condensation of L-serine with palmytoyl-coenzyme A in the initial and rate-limiting step in the *de-novo* synthesis of sphingolipids [18]. HSN1-associated SPTLC1 variants result in a shift of substrate specificity from L-serine to L-alanine and L-glycine resulting in the production of toxic deoxysphingolipids (DSBs) which are toxic to sensory nerves [17].

Clinical phenotyping of patients is a valuable tool in determining the pathogenicity of novel variants in known genes. It was therefore a surprise to find a recent study by Johnson et al. linking *de-novo* variants in *SPTLC1* to juvenile-onset amyotrophic lateral sclerosis (ALS) [2]. These patients presented in early childhood with generalised limb weakness and atrophy, bulbar disturbance, tongue fasciculations, and hyperreflexia, and were diagnosed with ALS.

Subsequent screening of cohorts of over 6000 patients with adult-onset ALS identified 20 novel *SPTLC1* variants in 23 patients (0.4%) with no sensory involvement. Murasel et al. also identified four variants in *SPTLC1* in 11 patients with juvenile-onset ALS. Unlike HSN1, these missense variants disrupted the regulation of SPT leading to overactivity and elevated levels of downstream SPT metabolites [3] in contrast to the SPT amino acid switch from serine resulting in accumulation of DSBs which leads to HSN1 [3].

These recent discoveries highlight the increasing complexity of gene identification in CMT and related disorders. In the case of *SPTLC1*, we now have two diseases, both due to missense mutations; one in which elevated DSB levels result in a sensory neuropathy and the other in which SPT overactivity leads to motor neurone disease.

## CMT1A

CMT1A is the commonest subtype of CMT accounting for almost 80% of cases of demyelinating CMT. It results from a duplication of the short arm of chromosome 17p containing the PMP22 gene. A deletion of the same region of chromosome 17p leads to hereditary neuropathy with liability to pressure palsies (HNPP) [14]. Peripheral nerves are therefore particularly sensitive to changes in PMP22 gene dosage and an obvious therapeutic strategy for CMT1A would be to reduce PMP22 gene expression. Confirmation that overexpression of PMP22 is key to the pathogenic process driving CMT1A has been proven from clinical studies, demonstrating that children born to one parent with CMT1A and one with HNPP, carrying a duplication on one allele and no PMP22 gene on the other allele have no phenotype (PMID 19,513,300) [19].

Initial strategies to reduce *PMP22* expression have focused on increasing cyclic adenosine monophosphate (cAMP) expression in Schwann cells which acts on a silencing element known to downregulate *PMP22* transcription [20]. Although several modulators of cAMP expression have been shown to be of benefit in animal models of CMT1A (e.g., Onapristone), their use has been limited by toxicity [21]. Ascorbic acid, a modulator of cAMP expression, has been shown to be effective in a single *in-vivo* study, but since then several double-blind randomised placebo-controlled trials have failed to show a therapeutic effect [22]. Much has been learnt about clinical trial design in CMT1A from these studies, not the least of which is the unsuitability of certain clinical outcome measures such as the ONLS and CMTNS version 1 for detecting clinical progression in CMT1A over a 2-year period. PXT3003, a combination drug of baclofen, naltrexone and D-sorbitol, two components of which also act through cAMP, in a preclinical and phase 2 study showed efficacy in CMT1A [23] leading to a double-blind phase 3 study. The results of this study were published earlier this year raising hopes for the large numbers of patients and families with CMT1A. Although the study reported an improvement in the ONLS in the high-dose group, the higher dose formulation was unstable, and by default, the patients were un-blinded to the intervention [24]. A further phase 3 study started recruiting in March of this year.

## CMT1A and PMP22 gene silencing

A promising therapeutic strategy for CMT1A is the use of synthetic DNA (antisense oligonucleotides) and silencing RNA molecules to suppress the expression of PMP22 RNA. This is a strategy that has already entered clinical practice with the widespread use of the ASO (inotersen) and siRNA (patisaran) to reduce TTR transcription [25, 26]. CMT1A is a gene dosage disorder and theoretically lends itself to a similar genetic approach aiming to reduce total PMP22 transcript levels. Unlike ATTR, however, it poses additional technical issues. First, a PMP22 silencing strategy poses a risk of developing HNPP if PMP22 levels are suppressed by more than 33%. In addition, PMP22 expression is confined to Schwann cells and for gene silencing therapy to be effective, the ASO or SiRNA must be able to penetrate the Schwann cell. A previous study of a PMP22 ASO in two murine models of CMT1A by subcutaneous injection demonstrated an effective suppression of PMP22 levels associated with an improvement in behavioural and electrophysiological parameters [27]. Further attempts to pursue this ASO have not been forthcoming, in the public sphere at least. PMP22 siRNA conjugated to a small natural lipid (squalene) to form nanoparticles used to suppress PMP22 transcript levels, represents a glimmer of hope in the field. Naked siRNA molecules have a short plasma half-life and are hydrophilic, impairing their delivery to Schwann cells. Viral vectors offer one approach to overcome this, but are expensive to produce and have cytotoxic and potential off target effects which although minimal may tip the risk–benefit balance for a slowly progressive disease such as CMT1A. The technology has already shown promise for the delivery of siRNA molecules to prostate and thyroid cancers. In a recent study by Boutary et al., 3-weekly intravenous injection of PMP22 siRNA nanoparticles improved behavioural and electrophysiological parameters in two mouse models of CMT1A in which they were able to demonstrate penetration of nanoparticles into Schwann cells [6]. The therapeutic effect was not seen in experiments using non-conjugated (naked) SiRNA. Interestingly, an effect was seen acutely that tailed off after 3 weeks, but could be maintained with regular administration. This short-lived effect may be an advantage for CMT1A where oversuppression of PMP22 may be deleterious.

## ATTR and gene editing

A further exciting development in the field of hereditary neuropathy has been the publication of the first clinical study of the use of CRISPR-Cas9 gene editing to treat a human genetic disease (Hereditary Transthyretin amyloidosis, also known as hATTR) [7]. hATTR is a fatal disease if untreated affecting many organs but principally the heart and peripheral nervous system. Therapeutic strategies for hATTR aim to suppress total TTR levels (both mutant and wild type) to unrecordable levels to prevent the accumulation of amyloid. There are currently two licenced drugs for hATTR, the ASO Inotersen and the lipid-conjugated siRNA, Patisiran. These two treatments have transformed the management of hATTR but require weekly or three weekly injections [25, 26].

Specifically, inotersen is an antisense oligonucleotide which is complementary to the sequence of TTR pre-mRNA. It is a 2’-O-methoxyethyl-modified RNA molecule which binds to the TTR pre-mRNA. This induces the endonuclease enzyme RNAse H to break down the mRNA–ASO complex resulting in suppressed expression of mutant and wild-type TTR protein [25]. Side effects include thrombocytopenia and glomerulonephritis [25].

Patisiran is a double-stranded, lipid-conjugated, synthetic small interfering ribonucleic acid molecule which acts similarly to RNA interference (RNAi), an endogenous mechanism for controlling RNA virus gene expression [24]. Patisiran targets hepatocytes by binding and activating the RNA-induced silencing complex which separates the siRNA duplex into single-stranded RNAs. This results in reduced production of mutant and wild-type TTR as the antisense siRNA targets the 3’ untranslated region of TTR mRNA. It can commonly lead to infusion reactions, thus rendering patients reliant on long-term pre-infusion treatment with steroids and antihistamines [7].

The majority of circulating TTR is produced in the liver and the holy grail in the treatment of hATTR (and many other genetic disease) would be to correct the genetic error at a somatic (or DNA) level, meaning that ongoing treatments would not be required. CRISPR Cas-9 gene editing technology is able to modify somatic DNA and therefore offers a therapeutic strategy of removing the TTR gene from its main site of production, the liver. In a recent open label study of six patients with ATTR and polyneuropathy, lipid nanoparticle (intravenous delivery) of mRNA for Cas9 protein and a single-guide RNA targeting TTR (NTLA-2001) led to a dose-dependent reduction in TTR of up to 87% at the highest dose [7]. ATTR is particularly suited to this treatment as unlike other dominant genetic diseases, the most effective treatment requires full suppression of both wild-type and mutant TTR. Nevertheless, this is one of the first applications of CRISPR Cas-9 gene editing to treat a fatal genetic disease. Current gene silencing therapies are costly and require life-long treatment. This approach offers the same or potentially improved benefit with only a single infusion.

## Conclusion

A significant proportion of patients with hereditary neuropathy remain genetically undiagnosed despite screening for all known gene mutations. It has always been assumed that this subgroup of patients would have private or uncommon gene mutations affecting a handful of families; however, the recent discovery of the CANVAS repeat expansion and the common recessive variant in SORD suggests that there are yet common but genetically hidden genes still to be found [1, 28]. Of perhaps more relevance to patients and clinicians is the emergency of new therapies for hereditary neuropathies, from small molecule drugs for metabolic neuropathies such as HSN1 and SORDD through to gene silencing and editing approaches for CMT1A and hATTR. This research was also supported by the National Institute for Health Research University College London Hospitals Biomedical Research Centre. AMR is grateful to the BMA for the Vera Down Award and to the National Institute for Health Research University College London Hospitals Biomedical Research Centre.
